# Serum levels of Cartilage Oligomeric Matrix Protein (COMP) increase temporarily after physical exercise in patients with knee osteoarthritis

**DOI:** 10.1186/1471-2474-7-98

**Published:** 2006-12-07

**Authors:** Maria LE Andersson, Carina A Thorstensson, Ewa M Roos, Ingemar F Petersson, Dick Heinegård, Tore Saxne

**Affiliations:** 1 Spenshult Hospital for Rheumatic Diseases, Halmstad, Sweden; 2 Department of Rheumatology, Clinical Sciences Lund, Lund University, Lund, Sweden; 3 Department of Orthopaedics, Clinical Sciences Lund, Lund University, Lund, Sweden; 4 Department of Experimental Medical Science, Section for Cell and Matrix Biology, Lund University, Sweden

## Abstract

**Background:**

COMP (Cartilage oligomeric matrix protein) is a matrix protein, which is currently studied as a potential serum marker for cartilage processes in osteoarthritis (OA). The influence of physical exercise on serum COMP is not fully elucidated.

The objective of the present study was to monitor serum levels of COMP during a randomised controlled trial of physical exercise vs. standardised rest in individuals with symptomatic and radiographic knee OA.

**Methods:**

Blood samples were collected from 58 individuals at predefined time points before and after exercise or rest, one training group and one control group. The physical exercise consisted of a one-hour supervised session twice a week and daily home exercises. In a second supplementary study 7 individuals were subjected to the same exercise program and sampling of blood was performed at fixed intervals before, immediately after, 30 and 60 minutes after the exercise session and then with 60 minutes interval for another five hours after exercise to monitor the short-term changes of serum COMP. COMP was quantified with a sandwich-ELISA (AnaMar Medical, Lund, Sweden).

**Results:**

Before exercise or rest no significant differences in COMP levels were seen between the groups. After 60 minutes exercise serum COMP levels increased (p < 0.001). After 60 minutes of rest the serum levels decreased (p = 0.003). Median serum COMP values in samples obtained prior to exercise or rest at baseline and after 24 weeks did not change between start and end of the study. In the second study serum COMP was increased immediately after exercise (p = 0.018) and had decreased to baseline levels after 30 minutes.

**Conclusion:**

Serum COMP levels increased during exercise in individuals with knee OA, whereas levels decreased during rest. The increased serum COMP levels were normalized 30 minutes after exercise session, therefore we suggest that samples of blood for analysis of serum COMP should be drawn after at least 30 minutes rest in a seated position. No increase was seen after a six-week exercise program indicating that any effect of individualized supervised exercise on cartilage turnover is transient.

## Background

Osteoarthritis (OA) is a disorder of multifactorial origin, which involves articular cartilage, synovium, subchondral bone, ligaments and/or the neuromuscular apparatus [1]. OA causes disability, pain and reduced quality of life. Risk factors for occurrence and progression of knee OA include age, previous injury, increased body mass index (BMI), genetic factors and high workload [2]. Studies suggest that low quadriceps strength increases the risk of OA development in the tibiofemoral joint [3]. Exercise is recommended for treatment of OA [4]. Several studies have shown that increased muscle strength provides joint stability and reduces pain and disability [5-7]. It is not clear if physical exercise also affects the properties of joint cartilage in vivo [8]. However, a recent study using delayed gadolinium enhanced magnetic resonance imaging in patients at high risk of knee OA, suggested increased glycosaminoglycan content in cartilage after exercise [9]. In vitro studies have shown enhanced production of matrix components in cartilage explants during cyclic compression [10,11].

A possible way to identify tissue matrix processes in OA is by monitoring tissue proteins or their fragments released into serum [12,13]. Such biomarkers also represent potential tools for monitoring effects of treatment on the tissue [14]. One such potential biomarker is cartilage oligomeric matrix protein (COMP). COMP is a five-armed 435 kD non-collagenous protein primarily identified in cartilage [15,16]. COMP interacts with collagen and is suggested to have a role in regulating fibril assembly as well as a structural role for maintaining the mature collagen network [17]. Studies of experimental arthritis have been used to demonstrate that changes in serum levels of COMP reflect processes in cartilage [18,19]. Supportive evidence from human arthritis is also emerging. For instance, therapeutic interventions which aim at retarding joint damage, such as blockade of tumour necrosis factor- alpha in rheumatoid arthritis, normalize serum COMP levels [20]. In other studies, the feasibility of serum COMP as a prognostic indicator of future joint damage and as a marker of ongoing joint damage, e.g. in OA and rheumatoid arthritis, has been suggested [21-24]. Thus, although small amounts of COMP are present in other tissues i.e. tendon and synovium, the evidence in favour of serum COMP being primarily derived from cartilage is compelling.

The objective of the present intervention was to monitor serum concentrations of COMP in a randomised controlled trial of physical exercise in patients with symptomatic and radiographically verified knee OA [25] to elucidate how standardised physical activity influences the turnover of COMP, study 1. In a supplementary small study we examined the short-term changes of serum-COMP immediately after exercise, study 2.

## Methods

### Subjects

In study 1 patients aged 36–65 years with symptomatic radiographically verified knee OA, uni- or bilateral Kellgren-Lawrence grade 3 or more, were included in a randomised controlled trial of physical exercise in knee OA. The clinical results of this study have previously been reported [25]. Patients were recruited from the Department of Radiology at the Halmstad County Hospital in Sweden. They had been referred to radiographic knee joint examination due to knee pain by their general practitioner.

Patients with inflammatory joint disorders, previous anterior cruciate ligament injury, or known injury to the menisci were excluded. Furthermore, patients with hip symptoms more severe than the knee symptoms (verified by clinical examination), patients being on the waiting list for knee replacement expected to be operated within 6 months, or with co-morbidities not allowing exercise were also excluded. Fifty-eight subjects fulfilled the criteria for inclusion and were randomised to exercise (n = 29,15 men and 14 women, with median (range) age 55 years (36–64) and median (range) Body Mass Index (BMI) 28.7 (23.0–43.0)) or rest (n = 29, 14 men and 15 women with median (range) age 57 years (46–65) and median (range) BMI 27.9 (22.0–45.0). The exercise group and the control group did not differ regarding age or BMI.

**Figure 1:**
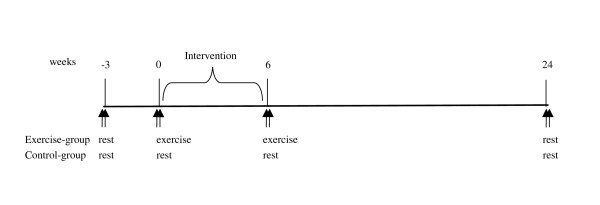
**Study design**. Blood-sampling procedures during the study from study baseline at -3 weeks to the end of the study at 24 weeks. Blood samples were obtained before and after 60 minutes exercise/rest at each occasion. The arrows indicates blood-sampling occasions. Two blood-samples at each occasions, with an hour apart.

Blood samples were obtained from all subjects at predefined intervals. At each occasion blood samples were obtained twice with an hour apart, in total 8 samples during study 1. Samples were taken at four different occasions, before intervention at -3 weeks, during intervention at two time points, 0 and 6 weeks, and after intervention at 24 weeks. At weeks -3 and 24 both groups were resting for one hour and at weeks 0 and 6 the exercise group was exercising and the control group was resting one hour between sampling of blood. The sampling schedule is outlined in figure 1.

All patients in the exercise group followed the exercise protocol. The program consisted of weight-bearing exercises aimed at increasing postural control, endurance and strength in the lower extremity. Exercises were performed at five stations at intensity of ≥ 60% of maximum heart rate (HRmax). This one-hour supervised, high intensity session was performed twice a week increasing aerobic capacity and muscular endurance and strength in the lower extremities. Home exercises for at least 30 minutes every day were prescribed for the other days during the six-week intervention period. The protocol has been described in detail [25].

The control group had no restrictions and were instructed to continue with their usual daily activities. All patients had been travelling to the hospital and there was no standardised rest period before the first serum sample was drawn. The control group was resting in a chair for one hour between the blood sampling procedures, figure 1.

We also designed a second, supplementary study to monitor serum COMP with repeated measurements after the exercise session. Seven patients, who had participated in the exercise-group in the first study, median (range) age 55 years (51–67) and median (range) BMI 27.2 (24.9–37.5) were included. The physical exercise consisted of a one-hour supervised, high intensity session, as performed in study 1. Blood samples were collected before, immediately after, 30 and 60 minutes after the exercise session and then with 60 minutes interval for another five hours. The patients were told not to exercise 24 hours before sampling. After the training session they were resting in a chair in the hospital waiting room until the last serum sample had been retrieved.

### COMP analyses

Venous blood samples were obtained from vena mediana cubitii. After clotting for 60 minutes at room temperature, they were centrifuged at 2000 g for 10 minutes at +4°C. The serum samples were stored at -20°C until all the serum samples were obtained. The samples were then stored at -80°C until analysis. Serum COMP levels were analysed with a sandwich-ELISA (AnaMar Medical, Lund, Sweden).

### Statistical methods and ethical approval

Comparisons between groups and within groups were performed using Mann-Whitney U test and Wilcoxon matched pairs test, respectively. A p-value of <0.05 was considered significant. Informed consent was obtained from the patients participating in the study. Ethical approval was obtained from the Research Ethics Committee, Lund University, Sweden (LU 99–98) and is in compliance with Helsinki Declaration.

## Results

### Study 1

#### 1. Serum COMP at start of study

**Figure 2:**
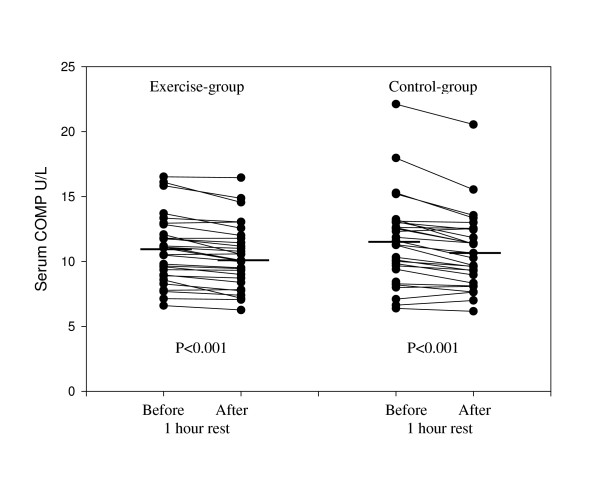
**Serum COMP at start of study**. Serum concentration of COMP at week -3, before intervention, in study 1. Each line represents an individual patient. Horizontal bars show median values for each group.

At time point -3 weeks, i.e. three weeks before the intervention started, there were no significant differences in serum COMP levels between the exercise and control group. Median (range) serum COMP levels were in the exercise-group 11.03 (6.60–16.52) U/L and in the control-group 11.29 (6.38–22.11) U/L. In both groups there was a slight decrease in serum-COMP levels after one hour of rest (p < 0.001), figure 2.

#### 2. Serum COMP during intervention

**Figure 3:**
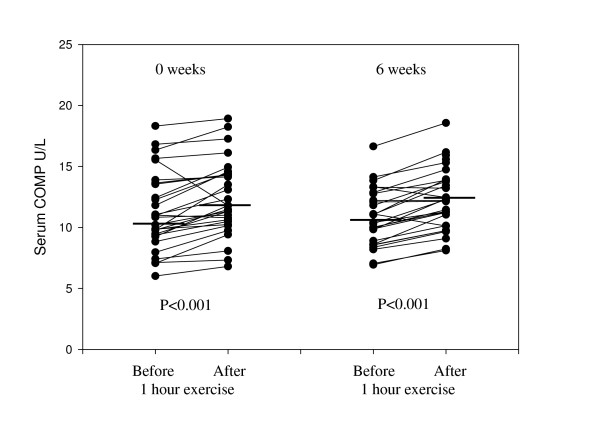
**Serum COMP during intervention**. Serum concentrations of COMP at weeks 0 and 6 in the exercise group in study 1. Each line represents an individual patient. Horizontal bars show median values for each sampling occasion.

Before exercise no significant differences in COMP levels were seen between the two groups at 0 or at 6 weeks, data not shown. After 60 minutes of exercise serum COMP levels increased (median increase 1.3 U/L, p < 0.001) at both time points in the exercise group, figure 3. At time point 0 and 6 the serum levels decreased slightly after one hour rest in the control group (median decrease 0.6 U/L, p = 0.003 and p = 0.053), data not shown.

#### 3. Serum COMP during the study period and at follow up

At time point 24 weeks, serum COMP levels decreased after one hour of rest in both groups, p = 0.002 in exercise group and p = 0.004 in control group, in a similar manner as at time point – 3 weeks. Serum COMP values did not differ between start (-3 weeks) and end of the study (24 weeks) in either group (data not shown). Thus the levels returned to baseline after the dynamic exercise intervention and after resting. Median (range) serum COMP levels were at the end of the study 10.92 (7.43–15.98) U/L in the exercise-group and 11.24 (7.28–22.00) U/L in the control group. The levels did not differ between the groups.

### Study 2

#### Serum COMP after standardised exercise in seven patients recruited from the exercise group in study 1

**Figure 4:**
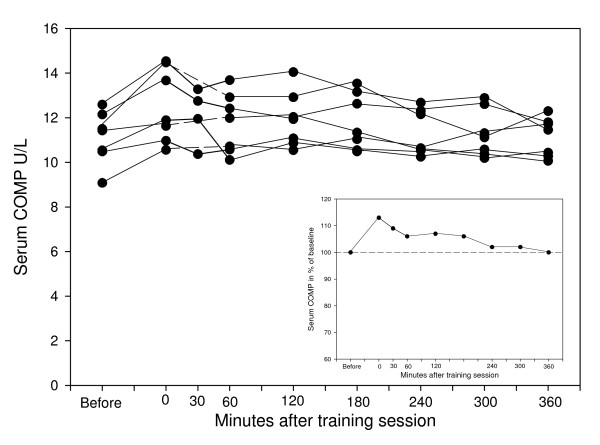
**Serum COMP after standardised exercise in seven patients recruited from the exercise group in study 1**. Serum COMP concentrations after a one-hour supervised exercise session in 7 patients, study 2. Each line represents an individual patient. The inserted figure shows serum COMP concentration in percent of baseline after a one-hour supervised exercise session.

Blood samples were collected before, immediately after, 30 and 60 minutes after the exercise session and then with 60 minutes interval for another five hours. The serum COMP levels increased immediately after exercise from a median level of 10.5 U/L to a median level of 11.6 U/L, p = 0.018, figure 4. The levels then decreased and at 30 minutes after the exercise session the levels did not differ significantly from baseline. As seen in figure 4 insert, the median level tended to decrease towards baseline levels during the whole observation period.

## Discussion

In this study of patients with knee OA, we could in a reproducible fashion show that physical activity according to a predefined protocol resulted in a significant increase in the serum levels of COMP. In contrast, resting in a chair for one hour reduced the serum levels slightly, but significantly. We did not observe any changes of the baseline COMP levels over the 24-week period, thus there appears to be no long-term influence on the turnover of COMP induced by this physical exercise. To further examine the changes in serum COMP after exercise we performed a supplementary study with more frequent measurements to elucidate the kinetics of serum COMP during the time immediately following the exercise session. We found that the serum concentrations rapidly returned to baseline, figure 4, and the concentrations did not differ from baseline after 30-minutes rest.

Recently Mündermann and co-workers reported that moderate walking activity during 30 minutes in healthy individuals significantly increased serum COMP as measured by the same assay as the one used in our study [26]. Importantly, in line with the results in the OA patients in the present study, they found that the serum levels had returned to baseline after 30 minutes. They also found that serum COMP decreased during rest in these individuals, also in line with the results of the present study.

Neidhart and co-workers found increasing serum levels of COMP using another assay in 8 athletes during a marathon run. In these endurance-trained runners serum COMP was at baseline elevated compared to healthy age- and sex-matched controls. The serum levels returned to baseline within 24–48 hours, which was considerably slower than in our study possibly because of more extreme exercise in these physically fit individuals [27].

One possible reason for the changes in serum COMP during physical exercise is mobilization of COMP from cartilage or other pressure loaded tissues. Increased cartilage degradation without compensatory increased synthesis cannot be ruled out. However, since the changes are transient it is unlikely that the exercise exerts any negative long-term effects on cartilage.

Another, perhaps more likely reason for the changes could be modifications of the extra-cartilage turnover of COMP with more of the protein being transported from the synovial fluid into the lymphatics and further into the blood stream during and immediately after exercise.

We have in another study shown a diurnal variation of serum COMP with stable levels during daytime and with the lowest levels found at night during bed rest. This is in line with the results of the present study where decreasing levels were found during rest [28]. In the study of diurnal variation a putitative half-life of COMP in serum was calculated to be 7.4 hours.

All patients had been travelling to the hospital and there was no standardised rest period before the first serum sample was drawn. This could mean that the observed decrease of serum levels during rest was due to a return to baseline after physical activity.

## Conclusion

The changes in serum levels of COMP, are small and do not compromise the utility of COMP as a biomarker, e.g. for monitoring treatment effects on the tissue. Thus, we suggest that samples of blood for analysis of serum COMP should be drawn after at least 30 minutes rest in a seated position. This is particularly important if sequential samples are to be obtained from the same patient. It should be noted that the information provided in this study pertains to patients with knee OA. However, since similar findings were reported for healthy controls it seems reasonable to adhere to this recommendation for all patients. Finally, it should be stressed that little information regarding the influence of exercise on serum concentrations for other putative biomarkers of cartilage is available. Thus, we suggest that similar studies should be performed for other markers.

## Abbreviations

COMP Cartilage oligomeric matrix protein

ELISA Enzyme-linked immunosorbent assay

OA Osteoarthritis

BMI Body mass index

HRmax Maximum heart rate

## Competing interests

DH and TS are cofounders and shareholders in AnaMar Medical and IP is a board member of AnaMar Medical.

## Authors' contributions

MA took part in the design of this study, in the blood sampling, carried out the COMP-analyse, performed the statistical analysis and drafted the manuscript.

CT participated in the design of the study, coordinated the clinical parts and carried out the physical training.

ER participated in the design and coordination of the study.

IP participated in the design of the study, helped in the statistical discussions and helped drafting the manuscript.

DH participated in the design of the study and assisted in drafting the manuscript.

TS participated in the design of the study, helped in the statistical discussions and helped drafting the manuscript.

All authors read and approved the final manuscript

## Pre-publication history

The pre-publication history for this paper can be accessed here:
